# Exploring the Role of Web-Based Interventions in the Self-management of Dementia: Systematic Review and Narrative Synthesis

**DOI:** 10.2196/26551

**Published:** 2021-07-26

**Authors:** Abigail Rebecca Lee, Esther Vera Gerritzen, Orii McDermott, Martin Orrell

**Affiliations:** 1 Mental Health and Clinical Neurosciences School of Medicine University of Nottingham Nottingham United Kingdom

**Keywords:** systematic review, narrative synthesis, dementia, web, app, online, mobile phone, self-management

## Abstract

**Background:**

The increasing prevalence of dementia has promoted a move toward equipping people with the skills required for greater self-management of the condition to enable a better quality of life. Self-management encompasses numerous skills, such as goal setting and decision making, which aim to improve an individual’s physical and mental well-being when they live with long-term health conditions. Effective self-management may lead to increased well-being and quality of life. Reviews of web-based and app-based interventions have suggested that they have the potential to provide self-management support for people living with a range of conditions, including dementia.

**Objective:**

The aim of this review is to explore the existing use of web-based or app-based interventions that facilitate or support self-management in dementia and discuss their effectiveness in promoting self-management and independence.

**Methods:**

A total of 5 electronic databases were systematically searched for relevant articles published between January 2010 and March 2020. Included studies were appraised using the Downs and Black checklist and the Critical Appraisal Skills Program qualitative research checklist. A narrative synthesis framework was applied using tables and conceptual mapping to explore the relationships within and among studies.

**Results:**

A total of 2561 articles were identified from the initial search, of which 11 (0.43%) met the inclusion criteria for the final analysis. These included 5 quantitative, 4 mixed methods, and 2 qualitative studies. All the included articles were of fair to high quality across the two appraisal measures. Interventions were delivered through a range of web-based and app-based technologies and targeted several self-management concepts. However, there was inconsistency regarding the domains, often affected by dementia, that were targeted by the interventions reviewed.

**Conclusions:**

Web-based and app-based interventions for dementia can be delivered through a range of means and can target different aspects of self-management. The small number of studies included in this review report positive outcomes that seem to support the use of these interventions for people living with dementia. However, there is a clear need for more high-quality research into this type of intervention delivery and for studies that use a much larger number of participants across the dementia spectrum. Future research should consider the barriers to and facilitators of intervention adoption highlighted in this review and whether interventions can encompass the physical, social, cognitive, and emotional domains affected by dementia.

## Introduction

### Background

Technology-based interventions have the potential to provide practical and effective delivery of support to affected populations across a range of health conditions [1]. Their role in dementia care is still emerging, and more research is needed to explore their current use and potential impact and highlight gaps in the literature and knowledge [2].

Dementia currently affects an estimated 885,000 people in the United Kingdom [3,4] and leads to impaired ability and performance across multiple cognitive domains, such as memory, cognitive ability, and communication, which appear even in the early stages [4]. The provision of care for people aged above 65 years who are living with dementia currently costs the United Kingdom £34.7 (US $47.9) billion a year and is expected to rise to £94.1 (US $129.9) billion by 2040 [5]. With the ever-increasing aging population, it is estimated that 1.6 million people will have a dementia diagnosis by 2040 [5]. Social care costs alone amount to £15.7 (US $21.6) billion, and the hours of unpaid care by families equate to £13.9 (US $19.2) billion a year [5]. Enabling people living with dementia to manage their condition more effectively, improve their overall well-being, and maintain their independence for as long as possible may provide benefits for both the population living with dementia and the health and social care sectors [6]. The role of technology-based interventions in dementia care is still emerging; however, they may offer the potential to provide practical and effective delivery of support for people living with dementia and their families [6].

The UK government highlighted the importance of enabling people with dementia to live well and independently in their dementia action plan [7]. Self-management was identified as a potential strategy in response to the increasing incidence and prevalence of dementia and in helping people and their families to retain control over their lives. Self-management encompasses multiple components that can support an individual to improve their physical and mental well-being, either independently or in collaboration with their health care team [8]. These components include goal setting, decision making, problem solving, accessing and using resources, strong collaboration between patients and health professionals, and patient activation [8-10]. The latter refers to the knowledge, skills, and confidence an individual has in managing their long-term condition and overall health and has been linked to a lower number of medical appointments and hospital admissions [11].

The lived experiences of people with dementia vary considerably, and it has been suggested that this may be due to the interaction between cognitive impairment and a range of psychological and social factors [12]. One review found that multicomponent, nonpharmacological interventions for people living with dementia had a positive effect on the activities of daily living, cognitive functioning, and mood [13]. In addition, interventions targeted at dyads were found to have positive effects on the quality of life of people with dementia and their caregivers. Oyebode and Parveen [12] extended the previous evidence by updating the evidence base to consider randomized controlled trials (RCTs), controlled studies, and reviews from 2008 to 2015. The 61 studies and reviews included covered the entire dementia care pathway, from community-dwelling people to residential care and end-of-life care, and considered interventions aimed at caregivers [12]. Many of the publications included discussed residential care, with a focus on managing the behavioral symptoms of dementia. The authors concluded that more research was needed into care within the community-dwelling dementia population and a greater focus on interventions that help to enrich the overall quality of life.

A review of web-based interventions that targeted support and education to informal caregivers found that they have potential benefits for both the supporter and the person with dementia [14]. A systematic search of the literature pertaining to RCTs of web-based interventions resulted in 17 studies. Interventions were found to be effective in decreasing symptoms of depression and anxiety in informal caregivers but failed to significantly reduce caregiver burden or improve quality of life. However, 6 studies demonstrated that caregiver interventions had the potential to positively improve the symptoms of depression and anxiety in caregivers and the quality of life of people with dementia. The review suggested that, when tailored to individuals and targeted at both caregivers and people with dementia, web-based interventions have the potential to improve the well-being and quality of life of all involved in informal dementia care.

Although the older population is generally perceived to have fewer technology skills, there is an emerging evidence base suggesting that technology plays a role in the self-management of dementia. In fact, it has been suggested that technology has five potential roles in dementia care [15]: facilitating declining cognition, enabling better performance of daily activities, ensuring safety, helping maintain active social involvement, and providing support and reassurance for informal caregivers. All these roles aim to assist people living with dementia to maintain their independence, improve their quality of life, and contribute to their self-management.

Research focused specifically on app-based interventions targeted at people living with dementia has also supported their use in the self-management of the condition. A study exploring the use of tablet computers and apps by people with mild dementia demonstrated that people were quickly able to learn how to use new technology and engage positively with the content of the apps [16]. The findings highlighted the importance of motivational benefits for people to incorporate new technology into their daily lives, such as improving their self-management and quality of life. Access to informal technology support to aid adoption was shown to be valued by people living with dementia and their families. However, consideration should be given to individualizing interventions to encourage engagement [16]. Several factors should be considered when creating and delivering self-management interventions (SMIs) in dementia to maximize their potential benefit and use.

Dementia is a chronic, progressive condition that affects multiple faculties in daily life [4]. The evidence base for self-management in dementia is limited, particularly regarding support for people living with mild dementia [8,17]. Therefore, an in-depth review of the current knowledge and use of interventions, particularly regarding the role of technology, is needed.

### Objectives

There are a range of nonpharmacological digital interventions that may be beneficial to people living with dementia, such as cognitive stimulation therapy [18]. However, the aim of this review is to explore the existing use of web- or app-based interventions that facilitate or support self-management in dementia, the concepts they target, and their effectiveness.

The findings are likely to be useful to health services and policy makers when considering how to include self-management in dementia and to researchers to help design better studies on the effectiveness of web- and app-based SMIs. This review could provide useful insights into the role of web- and app-based interventions in the self-management of dementia, and the findings should be considered in clinical practice. A protocol was written for this review but was not registered with PROSPERO [19].

## Methods

### Overview

Narrative synthesis is one approach to the systematic review and synthesis of findings from multiple studies and different methodologies. Although it allows for the inclusion of statistical data, the distinguishing characteristic of narrative synthesis is the use of a textual approach to summarize and describe findings to form a story from the included studies.

### Search Strategy

A systematic search was conducted across five electronic databases in February 2020: Cochrane (Central Register of Controlled Trials), Web of Science, PubMed, Scopus, and ProQuest (Science Database, Technology Collection, PsycArticles, and Social Science Database). After scoping the literature, a trial-and-error process was applied to explore search term combinations. With each combination, every third title and abstract were screened on the first two pages of results to determine whether they were relevant to the review questions. The key terms found were combined to create the final search: (web* OR online* OR computer* OR internet* OR app* OR smartphone*) AND (intervention* OR support*) AND (self-manag* OR independ*) AND (dement*). Independence was found to be a term often used in discussions about self-management; therefore, it was included in the final search. Terms such as *tablet* were excluded from the search because of their connotations with pharmacological interventions found during the initial scope of the literature. The search included research, journal, and review or evaluation articles, as it was thought that these would encompass novel research and evaluation studies. The date limits of January 2010 to March 2020 were placed on the search to encompass any prospective publications.

### Study Selection

The search results were imported into EndNote (Clarivate Analytics), and duplicates were removed. Each title and abstract were read twice and vetted by the primary reviewer (ARL), with the inclusion criteria acting as a guide to identify possible papers. A second reviewer (EVG) independently examined 5% of the total results to provide a consensus on the quality of the search. Potentially relevant references were imported into Rayyan [20], ready for a full-text review by the 2 reviewers (ARL and EVG). Each reviewer independently read the full texts twice before deciding whether to include or exclude the review. Any conflicts regarding the inclusion or exclusion of papers at any stage of the process were discussed by the two reviewers. A manual search of the references from the included papers was conducted for any suitable additions.

### Inclusion Criteria

The inclusion criteria were as follows:

- Participant population included adults aged 18 years or older, with a confirmed diagnosis of dementia.
- Participant population was community dwelling.
- Included a web- or app-based intervention aimed at improving self-management or independence for people living with dementia.
- Intervention was for independent or dyadic use (involvement from an informal supporter).
- Included RCTs or quasi-experimental, observational, qualitative, or mixed methods studies.
- Publication dates were between January 2010 and March 2020. These years were selected based on the definition of web-based interventions by Barak et al [21].

### Exclusion Criteria

The exclusion criteria were as follows:

- Protocol papers, opinion pieces, conference abstracts, scoping reviews, or systematic reviews.
- Interventions that were exclusively for supporters.
- Studies with a focus on care management and community-delivered interventions where the planning and coordination of dementia care was the focus [22].
- Published in a language other than English, and a translation was not available.

### Data Extraction

The principal reviewer (ARL) completed the data extraction using bespoke extraction forms based on the guidance of the Centre for Reviews and Dissemination for systematic reviews [23]. The data extraction forms were piloted before the review. A second independent review of the completed data extraction was provided by EVG. The following data items were extracted: (1) study information, (2) study characteristics, (3) population characteristics, (4) intervention, (5) outcome data, and (6) results.

### Quality Assessment and Risk of Bias in Individual Studies

The quality of studies assessed aspects such as the appropriateness of the study design, the potential risk of bias, and the quality of reporting. A total of 2 assessment tools were used: the modified Downs and Black [24] checklist, as used in Trac et al [25], to measure study quality for quantitative trials and the Critical Appraisal Skills Programme (CASP) checklist for qualitative research [26]. Mixed methods studies were assessed using both checklists.

The scoring system used for the modified Downs and Black checklist followed that outlined in the study by O’Connor et al [27], with 24-28 points regarded as *excellent*, 19-23 as *good*, 14-18 as *fair*, and less than 14 as *poor*. The 10-item CASP checklist had three response options: *meeting the criteria*, *unable to tell*, and *not meeting the criteria*. It was scored according to the method detailed in Stansfeld et al [28], with *meeting the criteria* given a score of 1 and *unable to tell* or *not meeting the criteria* given a score of 0. For the tenth item, which asks how valuable the research is and does not provide the response options, the principal reviewer decided whether to award a score of 1. The principal reviewer administered a scoring system in which a score of 4 or less was defined as *poor*, 5-7 as *moderate*, and 8 or above as *high*. These tools were selected as they are suitable for randomized, nonrandomized, and qualitative studies. They have also been used in previous narrative synthesis systematic reviews [28,29] and are recommended by the Centre for Reviews and Dissemination [23].

### Data Synthesis

Narrative synthesis allows for the inclusion of qualitative, quantitative, and mixed methods studies and for a systematic yet transparent review of results. Therefore, owing to the diverse selection of studies and review transparency, narrative synthesis was viewed as the most suitable option for this review.

Unlike more analytical approaches to literature reviews, such as meta-analyses, narrative synthesis does not rely on a rigorously tested structured technique [30]. Popay et al [30] created guidance and a framework of four interconnecting elements to improve the transparency of narrative synthesis reviews. This review applied the following guidance and framework:

- Developed a theory of how the intervention works, why, and for whom: a scoping of the relevant literature provided a greater understanding of the review topic, and the rationale for using web- or app-based interventions in dementia studies was considered. This stage guided the research questions, development of the search terms, and inclusion criteria for the review.
- Developed a preliminary synthesis of ﬁndings of included studies: data were extracted from each of the studies and tabulated. Descriptive summaries of the same features from each study were extracted and tabulated to help with the initial comparison. Studies were clustered according to the methodology: quantitative, qualitative, and mixed methods.
- Explored relationships within and between studies: concept mapping was used on the extracted data on study interventions to explore the similarities and differences between the studies and the factors that might have affected this.
- Assessed the robustness of the synthesis: two validity assessment tools were used in this study. Quantitative studies were assessed using the modified Downs and Black checklist [25], and qualitative studies were assessed using the CASP checklist [26]. Studies with mixed methodologies were assessed using both tools.

## Results

### Reviewing Process

**Figure 1:**
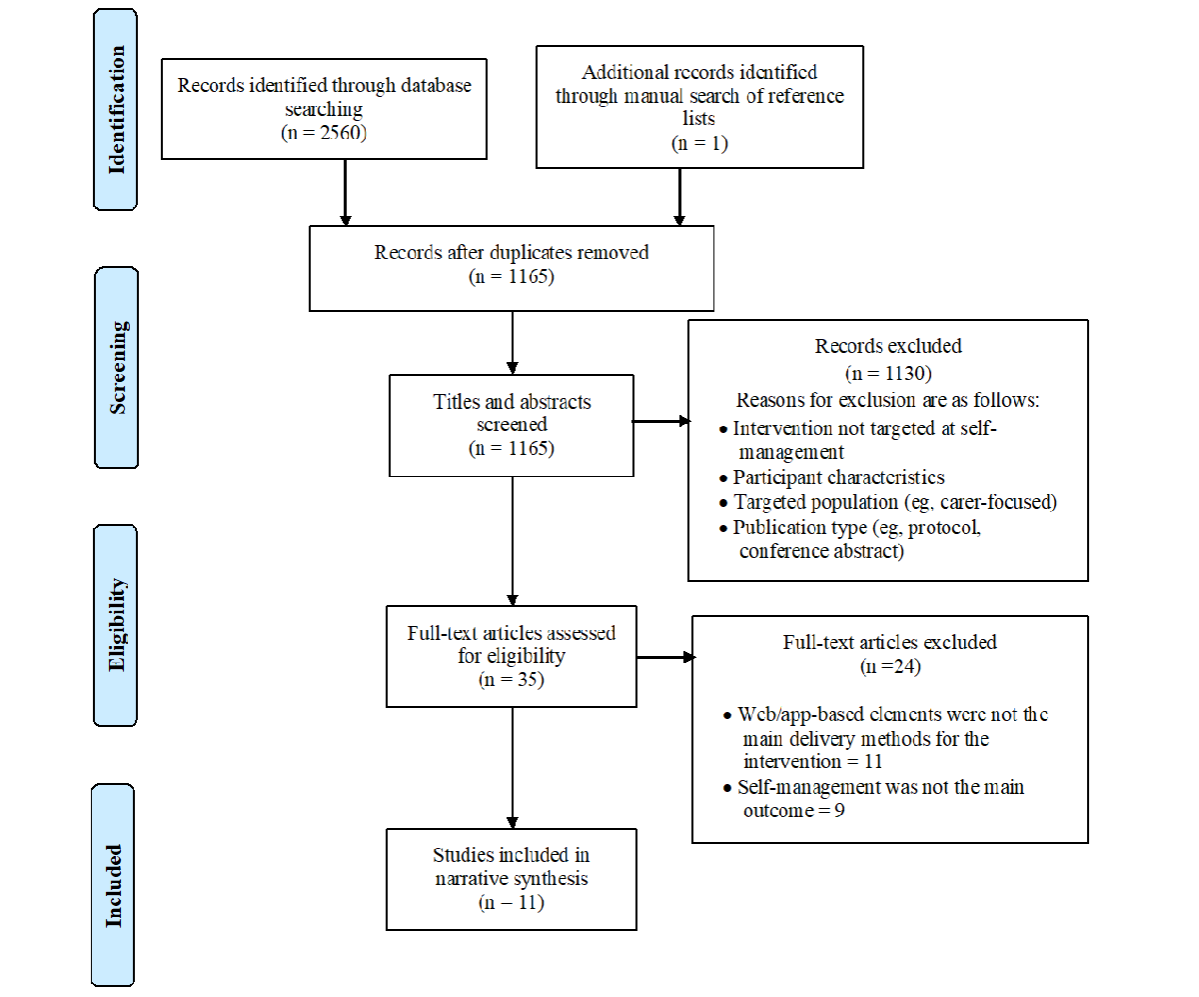
PRISMA (Preferred Reporting Items for Systematic Reviews and Meta-Analysis) diagram of the search and review process.

A total of 2560 references were identified using the search strategy. After duplicates were removed, 1164 references remained, and their titles and abstracts were screened for inclusion criteria. Of these, 1130 were excluded as they did not focus on relevant interventions or include participants with dementia, leaving 34 papers for full-text screening. One additional paper was found through a manual search of the reference lists of the papers selected for full-text screening. After a full-text review conducted by the principal and secondary reviewers, 11 papers met the inclusion criteria and were accepted for this review. The main reasons for exclusion were that the app- or web-based interventions were not the primary focus of the study; they were not described in sufficient detail for analysis, for example, lacking description of the intervention and mode of delivery; and the outcome measures were not relevant to self-management in relation to independence. Figure 1 shows a PRISMA (Preferred Reporting Items for Systematic Reviews and Meta-Analysis) diagram of the study selection process.

### Preliminary Synthesis of Findings

#### Study Characteristics

The included articles varied in location (Denmark=2, Sweden=1, United Kingdom=1, Netherlands=1, and United States=1); however, several studies did not specify a country (n=5). For studies with an unspecified location, the primary reviewer contacted the corresponding author but received no reply. Day and activity centers were the most common locations for the interventions (n=6), with private homes being the second most popular (n=5). Almost all the articles had either quantitative (n=5) or mixed methodology (n=4), and a nonrandomized, nonconcurrent multiple baseline approach was the most common study design (n=6). This meant that data from multiple baseline and intervention sessions were not collected simultaneously for all participants. No control groups or blinding procedures were used in any of the included studies.

#### Participant Characteristics

A total of 189 people living with dementia participated across the included studies, with an age range of 59-92 years. All studies had small sample sizes of ≤11, except for one study, which had 116 participants [31]. Alzheimer disease was the most common diagnosis among study participants (n=7), and the Mini Mental State Exam score was the most common measure used to describe participants (n=8). Scores varied from <6 to 22, indicating that the participants had mild to severe dementia. Participants were mainly recruited from day and activity centers for people living with dementia (n=6) or from memory clinics (n=3). Supporters were recruited in 4 studies, 2 as part of a dyad [32,33], and 2 as supporters [31,34]. Of the 121 supporters recruited, 119 (98.3%) were informal and 2 (1.7%) were formal (see Tables S1 and S2 of Multimedia Appendix 1 [31-41] and Multimedia Appendix 2 [31-41], respectively, for further details of the study and participant characteristics).

### Exploring Relationships Within and Among Studies

#### Robustness of the Synthesis

The quality of the included studies varied between fair and high. All the quantitative studies [35-39] were of fair quality, in accordance with the Downs and Black checklist scoring. These studies scored highly on reporting aims, intervention details, measuring outcome measures and providing a comprehensive summary of their findings. Mixed methods studies [31-33,40] scored high or moderate on the qualitative CASP checklist but fair on the quantitative checklist. Qualitative commentary on participant recruitment and the summary of findings complemented the quantitative reporting of aims, outcome measures, intervention details, and participant numbers and characteristics. Both measures suggested that greater reporting of data analyses, ethical considerations, the acknowledgment of monitoring for adverse events, and the inclusion of blinding would strengthen the methodology and study reporting. Of the 2 qualitative studies, one [34] scored highly on the CASP, whereas the other was moderate [41]. The reporting of study aims, data collection, and findings was strong; however, more details on the data analysis techniques used, reasoning for the chosen research design, and the relationship between researchers and participants would have been preferred. In addition, wider contribution of the research could have been discussed more thoroughly in both papers. The content of the included studies was judged to be of sufficient quality and robust enough to be included in the narrative synthesis. Table 1 shows the quality assessment scores of each of the included studies.

**Table 1:** Quality assessment scores.

Study	Methodology	Quality assessment score		Quality
		Value, n (%)	Total, N	
Perilli et al (2012) [35]	Quantitative	15 (54)	28	Fair
Perilli et al (2013) [36]	Quantitative	15 (54)	28	Fair
Lancioni et al (2017) [37]	Quantitative	16 (57)	28	Fair
Lancioni et al (2018) [38]	Quantitative	14 (50)	28	Fair
Lancioni et al (2019) [39]	Quantitative	14 (50)	28	Fair
Thorpe et al (2019) [32]	Mixed methods (quantitative)	14 (50)	28	Fair
Thorpe et al (2019) [32]	Mixed methods (qualitative)	7 (70)	10	Moderate
Øksnebjerg et al (2020) [31]	Mixed methods (quantitative)	14 (50)	28	Fair
Øksnebjerg et al (2020) [31]	Mixed methods (qualitative)	9 (90)	10	High
Kerssens et al (2015) [33]	Mixed methods (quantitative)	15 (54)	28	Fair
Kerssens et al (2015) [33]	Mixed methods (qualitative)	6 (60)	10	Moderate
McGoldrick et al (2019) [40]	Mixed methods (quantitative)	17 (61)	28	Fair
McGoldrick et al (2019) [40]	Mixed methods (qualitative)	9 (90)	10	High
Kerkhof et al (2019) [34]	Qualitative	8 (80)	10	High
Boman et al (2014) [41]	Qualitative	7 (70)	10	Moderate

#### Interventions

Concept mapping enabled a clear comparison of the interventions among the included studies. All the studies described their interventions in detail. There was a range of web- and app-based technologies used to deliver SMIs: touch screen computers (n=1), smartphone apps (n=3), and multicomponent (n=7). Of the multicomponent interventions, smartphones or tablets were the most commonly used (n=4), followed by earpieces or headphones (n=3) and headsets (n=3), although apps (n=2), computers (n=1), and smartwatches (n=1) were also used. These findings suggest that apps are becoming more popular in the delivery of interventions, either alone or as part of a more complex, multicomponent method. A total of 2 studies examined the same intervention but with different participants [35,36], and 3 others focused on a similar alternative intervention in different participant groups [37-39].

There were similarities and differences among the aims of the studies with regard to the self-management concepts targeted by the interventions. In total, 7 of the studies focused on interventions that targeted more than one self-management concept, although no intervention covered all domains or self-management concepts. One study targeted three concepts, 6 studies considered two concepts, and 3 studies focused on one self-management concept. Four overarching self-management concepts were widely assessed across the included studies: independence, activities of daily living, communication, and cognition.

**Figure 2:**
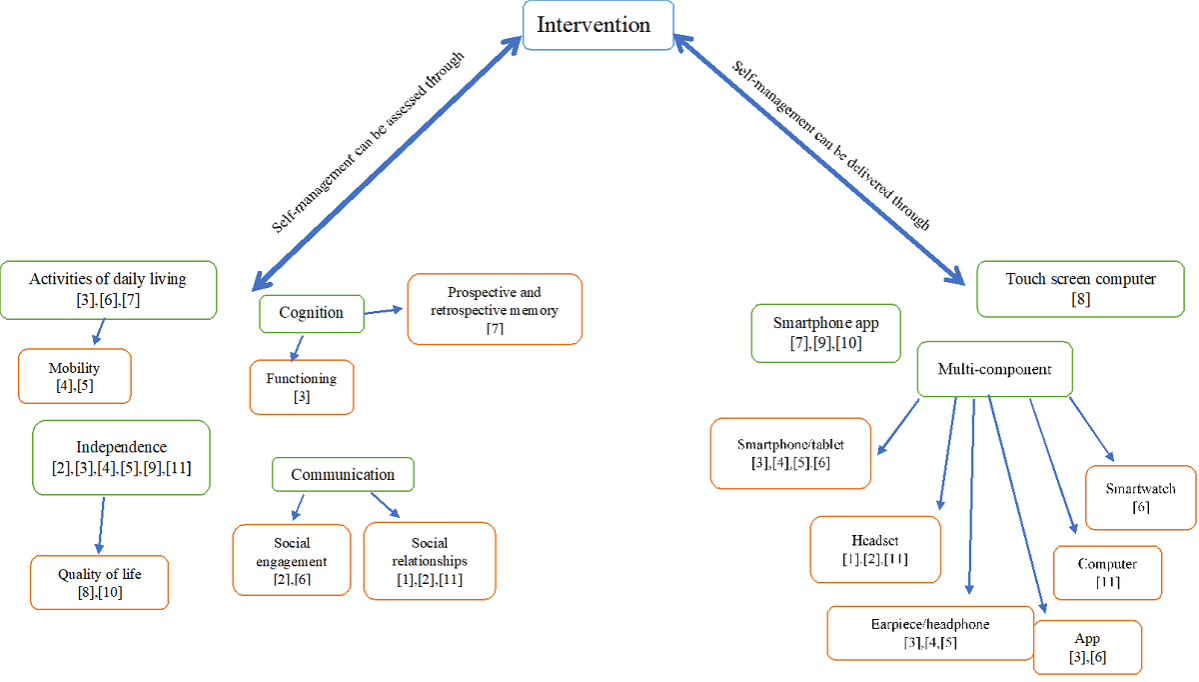
Concept map of self-management interventions. Numbers indicate the study reference number in tables.

Independence was the most commonly identified concept (n=8). A total of 2 studies focused on the effect of independence on the quality of life. Other popular concepts targeted by interventions in several studies were improving activities of daily living (n=5) and communication (n=5). Studies that explored communication could be divided into enhancing social relationships (n=3) and promoting social engagement (n=2). A total of 2 studies centered on improving cognitive functioning and memory enhancement. Figure 2 shows the intervention concept map. The numbers refer to the study identities found in the study and outcome tables.

All interventions could be tailored or modified in their delivery to fit individual needs or goals. The study period was reported either through the number of intervention sessions or the number of days, with one exception where the duration of the session was provided. The number of intervention sessions varied between 20 and 119 sessions and the number of days from 24 days to 9 months. Researchers or the research team was heavily involved in the intervention setup and provision across all 11 included studies.

#### Outcomes

Table S3 (Multimedia Appendix 3 [31-41]) outlines the outcomes and key findings of each of the included studies.

##### Activities

A total of 6 studies focused on outcomes that measured or explored the completion of activity. In total, 2 studies measured the completion rate of independent phone calls to people who were relevant to the participants [35,36]. The mean number of independent calls in the baseline of both studies was 0; however, this increased to around 4 during the respective interventions. A similar study explored the experiences of using a mock-up videophone [41]. Observations and qualitative feedback from participants showed that they initially struggled with the new intervention but could use it independently following guidance from the research team. Participants reported that the intervention was enjoyable to use, but they would have preferred more options to individualize it. A total of 3 studies had outcomes that measured independent ambulation and object use [37-39]. The interventions in these studies appeared to have a considerable impact on participants’ ability to start and complete independent activities successfully, such as making a cup of coffee or preparing food. In particular, one study reported a significant improvement in all participants executing the correct steps to complete their activities [38].

##### Engagement

The outcomes of the other 5 studies [31-34,40] explored the wider impact and experiences of app-based and wearable technology in dementia care. Interventions in 2 studies led to increased activity levels and a sense of independence in participants, which promoted positive engagement with daily activities [32,33]. Several issues regarding the incorporation of web- and app-based interventions in dementia were highlighted in the qualitative outcomes. Contextual and personal factors, such as a lack of confidence in using technology, concerns about dealing with technical difficulties, and forgetting to use apps, were some of the issues raised by participants and their families. These factors were key to nonadoption in the respective studies and should be considered when designing and delivering future studies in dementia care.

##### Adoption and Usability

The adoption and usability of apps were measured in 2 studies. One study [31] trialed the Rehabilitation in Alzheimer Disease Using Cognitive Support Technology app-based intervention, designed to assist with memory symptoms and daily activities. The overall mean Usefulness, Satisfaction, and Ease of use Questionnaire for dementia scores in this study of 40 for participants and 34 for supporters out of a total of 60 indicated a moderately high-level satisfaction rating of the intervention regarding usefulness, satisfaction, and ease of use. The researchers divided participants into adopters and nonadopters, depending on their usage of the intervention. There were 18 participants and 7 supporters who continued to use the app after the 90-day study period and were classed as *adopters*. However, 47 participants and 78 supporters did not activate the app. The survey, which was completed by 35 participants, showed that those who adopted the app were not significantly different from nonadopters in their skills, level of experience, and need for help when using a tablet [31]. For those who did not activate or continue to use Rehabilitation in Alzheimer Disease Using Cognitive Support Technology, several reasons were given, including that it was not relevant for the stage of their condition and a preference for using nontechnology-based solutions [31].

Another study [40] used the Unified Theory of Acceptance and Use of Technology Questionnaire to assess changes in attitudes toward the use of their reminder app in eight domains. Unified Theory of Acceptance and Use of Technology Questionnaire scores were collected from 2 participants, with one showing a positive decrease in pre- and postscores but the other showed a negative increase in half of the domains. The adoption of web- and app-based interventions appears to be dependent upon individuals connecting with the intervention and feeling confident about using it and may or may not result in a positive research outcome.

## Discussion

### Principal Findings

After reviewing the current evidence, it is clear that web- and app-based interventions have the potential to benefit the lives and care of people living with dementia. This narrative synthesis review examined the literature discussing the use of web- and app-based technology in delivering SMIs in dementia care. From the 11 studies that met the inclusion criteria, it is apparent that a range of methodologies have been applied when researching this topic. All the included studies were generally of fair to good quality, and the results were consistent and coherent, which suggests that the synthesis was robust. However, the scores from the quality appraisal measures suggest that there is a lack of high-quality research on web- and app-based interventions. More details on participant recruitment methods and the acknowledgment of potential adverse events were needed, and the blinding of those conducting outcome measures would have strengthened the methodology. The interventions reviewed targeted independence, communication, and activities of daily living, and 7 studies focused on multiple concepts of self-management. However, there was inconsistency regarding the number of domains related to dementia self-management, such as daily living activities, that were targeted by each intervention.

Most studies had very small participant numbers, ranging from 3 to 11, except for Øksnebjerg et al [31], who recruited 116 participants living with dementia and 98 supporters. Owing to the small sample sizes, studies were unable to conduct comprehensive analyses on their results and often relied on reporting changes in the mean scores of outcome measures. Recruitment methods across studies were open to bias, as they usually relied on people who had contact with memory clinics or day centers. Therefore, the participants might not have been representative of the wider dementia population. There were no suitable RCTs, and none of the included studies reported blinding participants or researchers. This highlights the shortfall in comprehensive, large-scale RCTs of web- and app-based SMIs in dementia and identifies an area for future research.

### Critical Reflection

Reflection was undertaken by the authors throughout the review process to identify any limitations or biases that could influence the review findings. As critical reflection is not a linear process, the authors acknowledge that there may be additional missed limitations. One strength of this review is that the search terms were created according to the scope of the relevant literature. This helped ensure that the final search would find the most relevant results and that the number of missed articles would be significantly reduced. Another strength is that the articles were differentiated and excluded using a standardized definition of care management. Having a definition meant a uniform exclusion of articles and a greater inclusion of self-management–focused results.

Although the search terms appear robust and the results were excluded in a uniform manner, this review has several limitations. First, the included articles were limited to those published in English or those that had an English translation available, which might have led to some relevant research being missed. It was decided to restrict participant populations to people living with dementia in the community, rather than those in residential homes or institutionalized care, which means the search strategy missed any web- or app-based SMIs in those settings. This could be a potential area for future reviews. Finally, owing to the small number of participants involved across the included studies, it is difficult for this review to provide a comprehensive evaluation of the effects of web- or app-based interventions on the self-management of dementia. There is a need for studies to explore these interventions in larger samples of people living with dementia and across a range of dementias and severities, for more significant conclusions to be drawn. As narrative synthesis takes a textual approach to analyzing evidence, the quality of methodological reporting could have biased the findings.

### Comparison With Previous Work

To our knowledge, this is the first review to systematically synthesize evidence concerning web- and app-based SMIs for people living with dementia. However, previous reviews have identified digital interventions aimed at people living with noncommunicable diseases, such as cardiovascular diseases. One such review examined the potential role of digital interventions in promoting healthy behavior change and improving self-management [1]. A search of 9 databases resulted in 29 publications meeting the inclusion criteria, with these studies covering 7 different interventions. All 7 interventions were identified as web-based, with 4 also having mobile-based delivery and targeted health behaviors such as physical activity and diet.

Clinical and psychosocial outcomes, such as quality of life, were reported in the included studies. Significant effects on psychosocial outcomes were reported only for one intervention. However, positive clinical outcomes on activity levels, disease-specific self-care, and self-monitoring behaviors were apparent across all interventions. These findings present a similar view to this review and indicate that evidence-based digital interventions, often provided through web- or app-based delivery, have the potential to promote positive behavior change and better support the self-management of conditions when delivered with correct guidance and tailored to the individual.

### Conclusions

This review explored and examined evidence concerning web- and app-based interventions targeted at self-management of dementia through a narrative synthesis methodology. Many of the interventions reviewed had a positive impact on the self-management concept they were targeting, which suggests that their use could prove beneficial in dementia care. The successful adoption of these interventions appears to be dependent on individuals’ engagement and their confidence in using the technology. Common factors influencing nonadoption appear to be a lack of confidence or familiarity with using technology, apprehension about encountering and resolving technological difficulties, and forgetting to use the intervention.

The findings are beneficial to health services and policy makers in considering how to incorporate self-management in dementia care and to researchers to help design better studies on the effectiveness of web- and app-based interventions. Barriers to adoption and implementation should be considered when delivering these interventions digitally to maximize the potential reach and effect on people living with dementia and their families. Conclusions drawn from this review will provide a positive contribution to the growing evidence base and increase the understanding of the use of these types of interventions in the self-management of dementia and their role in service provision.

## Appendix

Multimedia Appendix 1 Table S1. Details of study characteristics.

Multimedia Appendix 2 Table S2. Details of study interventions.

Multimedia Appendix 3 Table S3. Outcome measures and key findings from each included study.

## Abbreviations

CASP: Critical Appraisal Skills Program
PRISMA: Preferred Reporting Items for Systematic Reviews and Meta-Analysis
RCT: randomized controlled trial
SMI: self-management intervention
