# Supplementary material for: Exploring the Role of Web-Based Interventions in the Self-management of Dementia: Systematic Review and Narrative Synthesis
Source: J Med Internet Res. 2021 Jul 26;23(7):e26551. doi: 10.2196/26551 (PMC8367157; doi:10.2196/26551)
Supplement: Multimedia Appendix 1 [file jmir_v23i7e26551_app1.docx]

Table S1. Details of study characteristics.

| *Study*  *[Identity number]* | *Aims* | *Design* | *Duration* | *Setting* | *Participant demographics* | *Inclusion*  *criteria* | *Exclusion criteria* | *Dropouts* |
| --- | --- | --- | --- | --- | --- | --- | --- | --- |
|  |  |  |  |  |  |  |  |  |
| [1] Perilli et al., 2012 | To assess the effectiveness of a computer-aided telephone system in people with AD to make calls independently. | Non-randomized multiple baseline design | 2-5 baseline sessions and 3 familiarisation sessions. 50 intervention sessions. Intervention sessions lasted approximately 10mins. | Day centre. No country mentioned. | *Total =* 4  M/F = unknown  *Age range =* 73-83  *Moderate AD =* 4  MMSE = 13-18  Hamilton Depression Rating = 4-15  Recruited from a day centre. | - Considered to have moderate AD - Unable to use a telephone device independent - Able to understand verbal and visual instructions | None mentioned | None |
| [2] Perilli et al., 2013 | To carry out a social validation assessment of a computer-aided telephone system versus a conventional condition. | Non-randomized multiple baseline design | 3 or 5 baseline sessions and 5 familiarisation sessions. First group completed 20 intervention sessions, second group 50 sessions. Intervention sessions lasted approximately 10mins. | Day centre. No country mentioned. | *Total* = 5  F = 5  *Mean age =* 80 (range 73-89)  *Mild AD =* 2  *Moderate AD =* 3  MMSE = 14-22  Hamilton Depression Rating = 11-16  Recruited from day centre. | - Unable to use a telephone device independently - Able to understand verbal and visual instructions | None mentioned | None |
| [3] Lancioni et al., 2017 | To assess a technology-aided program to help people with mild to moderate AD carry out daily activities independently. | Non-randomized multiple baseline design | 3-5 baseline sessions and 3-4 familiarisation sessions. 34-78 intervention sessions. | Activity and care centres. No country mentioned. | *Total =* 8  M=1/F=7  *Age range =* 64-79  *Mild AD =* 4  MMSE = 21-25  *Moderate AD =* 4  MMSE = 15-22  Does not specify recruitment setting. | - Mild to moderate AD - Verbalise interest in using a device - Difficulties with daily activities | None mentioned | None |
| [4] Lancioni et al., 2018 | To assess the effectiveness of interventions to promote (a) independent start and accurate performance of daily activities and (b) supported ambulation. | Non-randomized multiple baseline design | *Study 1 –* 5-10 baseline sessions/37-82 intervention sessions. Each lasted 1.5-2hr.  *Study 2 –* 4-11 baseline sessions/73-119 intervention sessions. Each lasted 3 minutes and occurred 3 to 7 times a day. | Day centres. No country mentioned (ethics Italy). | *Study 1*  *Total =* 8  M=5/F=3  *Age range =* 73-92  MMSE = 16-24  *Study 2*  *Total =* 9  M=4/F=5  *Age range =* 70-92  MMSE = <6 - 14  Both groups recruited from centres attended by people with dementia. | *Study 1 –*   - Generally passive when left alone - Capable of following verbal activity reminders and instructions - Verbalized interest in using a program such as the one in the study   *Study 2 –*   - Unable to ambulate independently - Enjoyed stimulation events (e.g. music) - Ambulated in response to verbal prompts - Staff/families considered ambulation intervention relevant to participant | None mentioned | *Study 1 –* 2 due to lack of interest or poor health  *Study 2 –* 4 due to health or practical reasons (not included in participant numbers) |
| [5] Lancioni et al., 2019 | To assess a smartphone-based intervention to achieve goal-directed ambulation and object use in people with moderate AD. | Non-randomized multiple baseline design | 6 baseline sessions and 51-107 intervention sessions. | Day centres. No country mentioned. | *Total =* 11  M=5/F=6  *Mean age =* 83  *Moderate AD =* 11  MMSE =  11-19  Recruited from day centres. | - Unable to ambulate independently - Known to enjoy stimulation events - Able to ambulate to specific destinations if verbally encouraged - Had verbalised their willingness to participate - Staff/families considered ambulation intervention relevant to participant | None mentioned | None |
| [6] Thorpe et al., 2019 | To assess the feasibility of using smartphone and smartwatches to strengthen rehabilitation in early-stage dementia. | Mixed methods design | Participants used technology for at least 8 weeks. | Private homes. Denmark. | *Total =* 6 (each with caregiver)  M=4/F=2  *Age range =* 65-78  *Diagnosis =* mild-to-moderate dementia  MMSE =  23-27  Recruited from the dementia and memory clinic. | - Community dwelling with their primary caregiver - Early stage of dementia | - Any disability that affects use of devices or activity levels | *3* – enrolled but dropped out due to illness or feeling daunted about using the devices before start. |
| [7] Øksnebjerg et al., 2020 | To assess the applicability and usability of the ReACT app. | Mixed methods design | Intervention period 90 consecutive days after activating the app, or 90 days from study inclusion for participants who did not activate the app. | Private homes. Denmark. | *Total =* 116 people with dementia and 98 supporters  *Mean age =* 68  *AD =* 65  *Frontotemporal =* 3  *Vascular =* 2  *Lewy body =* 1  *MCI =* 9  *Other =* 27  *Unresolved =* 5  MMSE = 11-30  Recruited from 9 memory clinics. | - Patient at the memory clinics - Showed motivation to trial the app - Had access to a tablet computer | None mentioned | *4 -* excluded from original sample number due to insufficient background information |
| [8] Kerssens et al., 2015 | To assess the usability and adoption of a psychosocial touch screen interventions. | Mixed methods design | Baseline measures conducted at private homes. Intervention period was scheduled to last 3 weeks but ranged from 24-57 days. Follow-up interview/ assessments at end of intervention period. | Private homes. Georgia, USA. | *Total =* 7 dyads (people with dementia and supporter)  MMSE = 11-27  Recruited from retirement communities, Alzheimer’s Association, day centres and senior services centre | - Living independently in the community - Cohabitating couple or supporter dyads - Dementia diagnosis or assistive need in care recipients - Mild to moderate supporter distress - Aged 50+/supporter aged 21+ | - MMSE <10 - Dementia diagnosis in supporters - Severe supporter distress - Comorbid conditions that would compromise participation | *5 dyads –* excluded (not included in the 7 final dyads) because the person with dementia died (n=2), was transferred to a nursing home (n=1), repeated hospital admissions (n=1), or the supporter could not agree on the focus of the intervention (n=1). |
| [9] McGoldrick et al., 2019 | To assess the effectiveness and usability of a reminder tool on the MindMate app on prospective memory. | Mixed methods multiple baseline design | Baseline phase 5,6 or 7-weeks. Pre-intervention phase 1-3 weeks,  then MindMate for a 5-week period. Clinical interviews conducted pre-and post-study. | Private homes. Scotland, UK. | *Total =* 3  M=2/F=1  *Age range =* 59-74  *Mild AD =* 3  Recruited from community mental health teams | - Mild dementia diagnosis - Memory difficulties confirmed by professional or family member - Owned a smartphone or tablet with internet access - Had a partner willing to support and monitor memory aid use | - Pre-existing neurological or severe psychiatric condition - Moderate or severe dementia - Visual or auditory difficulties - Developmental learning disability - First language other than English - Currently using online or electronic memory aids | *1 –* withdrew during intervention due to experiencing technical difficulties but continued using baseline phase. Follow-up interview was held, and partner continued to record prospective memory forgetting. |
| [10] Kerkhof et al., 2019 | To develop an interactive web-based selection tool for self-management and meaningful activities in dementia. | Qualitative with user-participatory design | 9 months | Day centres. The Netherlands. | *Total =* 8 (+ 8 informal/2 formal supporters)  M=7/F=1  *Mean age =* 78.6  *AD =* 5  *Frontotemporal =* 2  *MCI =* 1  Recruited from two meeting centres and one day centre. | - Community-dwelling - Care dependent - Mild dementia (with/without confirmed diagnosis) | None mentioned | None |
| [11] Boman et al., 2014 | To assess the usability of a videophone mock-up for people with dementia and their significant others. | Qualitative case study design with interviews and observations | Interviews and observations at private homes lasted 1.5-2hrs. Intervention sessions lasted 2-3hrs. | Private homes & Living laboratory. Sweden. | *Total =* 4  M=2/F=2  Recruited through an investigation memory unit. | - Dementia diagnosis - Able to participate in interviews and observations - Willing to test intervention in a living laboratory - Have a significant other willing to participate | None mentioned | None |
