# Supplementary material for: Exploring the Role of Web-Based Interventions in the Self-management of Dementia: Systematic Review and Narrative Synthesis
Source: J Med Internet Res. 2021 Jul 26;23(7):e26551. doi: 10.2196/26551 (PMC8367157; doi:10.2196/26551)
Supplement: Multimedia Appendix 2 [file jmir_v23i7e26551_app2.docx]

Table S2. Details of study interventions.

| Study  [Identity number] | Components of intervention | How it’s self-management | Who provided/set up the intervention | Intervention delivery and format | Individualisation & modifications |
| --- | --- | --- | --- | --- | --- |
|  |  |  |  |  |  |
| [1] Perilli et al., 2012 | - Net-book computer, microswitch device to activate the computer, headset with microphone, and a software program - System presents identification names and photos of relevant people to call and responds to the microswitch activations to make calls | Ensuring people with dementia have an opportunity to communicate with others is instrumental in helping maintain their social relationships and reduce/slow down their social withdrawal | - Research assistant in baseline, familiarisation, and intervention sessions - Interrater reliability in 40% of intervention sessions (second RA) | - Baseline – participants provided with phones alone and told they could make phone calls - Familiarisation sessions – 3 - Intervention – 50 10-mins daily sessions, where the system was used independently. Computer presented 7-12 contacts and verbally gave the identification attributed to that photo. If they responded within 3-4mins, the computer activated a call. If not, it moved to the next photo | List of contacts and identification expressions were individualised |
| [2] Perilli et al., 2013 | - Net-book computer, microswitch device to activate the computer, headset with microphone, and a software program - System presents identification names and photos of relevant people to call and responds to the microswitch activations to make calls | Ensuring people with dementia have an opportunity to communicate with other is instrumental in helping maintain their social relationships independently, which can raise self-determination and social engagement | - Research assistant in baseline, familiarisation, and intervention sessions - Interrater reliability in 30-50% of intervention sessions (second RA) | - Baseline – participants provided with phones and told they could make calls - Familiarisation sessions – 5 - Intervention – either 20 or 50 regular daily sessions, where the system was used independently. Computer presented 8-12 contacts and verbally gave the identification attributed to that photo | List of contacts and identification expressions were individualised |
| [3] Lancioni et al., 2017 | - Tablet computer, with the Talking Alarm Clock app, and a Bluetooth earpiece - Earpiece provides instructions and encouragement during activities | Help people with dementia engage independently with daily living, by verbally scheduling activities. Possible benefits for their cognitive functioning, social image, and physical condition | - Research assistant in the baseline, familiarisation, and intervention sessions | - Baseline 1 – verbal and printed list of 6 or 7 activities executed and the times due - Baseline 2 – executed 6 or 7 activities, one at a time - Familiarisation – 3-4 sessions - Intervention – activities executed using tablet/earpiece set-up | The activities scheduled were personalised. |
| [4] Lancioni et al., 2018 | - Study 1 – Tablet or smartphone device with Android system and the Talking Alarm Clock app and a wireless Bluetooth earpiece - Study 2 – Tilt microswitch, notebook computer, and earpieces. Microswitch detected step responses, and the computer recorded these and provided stimulation events and verbal prompts | Both interventions promote completion of daily activities independently and support participants’ ambulation | - Research assistants in baseline and intervention sessions | *Study 1* –   - Participant provided with the wireless earpiece linked to the tablet or smartphone, which promoted independent start and correct performance of the activities   *Study 2* –   - Participants used walkers with the technology, which provided stimulation and prompts | Study 1 – The activities were adapted to participants’ characteristics in terms of steps and complexity  Study 2 - Stimulation events consisted of 5-s segments of old songs, religious hymns, and prayers, which were deemed preferred for the participants |
| [5] Lancioni et al., 2019 | - Smartphone with a light sensor and a variety of audio instruction files; headphones; three battery-powered light sources | Help manage mobility difficulties to increase independence and quality of life | - Research assistant | - Familiarisation – 3-6 sessions - Intervention - sessions lasted 3-5 mins and carried out 2-4 times a day. Smartphone presented instructions to bring an object to a specific destination. Instructions repeated at 10-15s intervals until they reached the destination. Once completed, 15s of stimulation (song/hymn/ comic sketch) was played. This was repeated for second and third objects | The stimulation played once a goal was completed was tailored to individual preferences |
| [6] Thorpe et al., 2019 | - Smartphone and/or smartwatch. The watch self-reports activity levels and a mobile app is used to get participants to evaluate their daily activity and mobility levels | Encourage people to remain active and socially engage through mobility and activity goals | - Research team | - Set-up support and manuals on device use were provided - Intervention - Participants set goals to follow and evaluated attainment in weekly phone calls. Mobile self-reports were issued daily to evaluate perceived activity and mobility levels for that day - After, semi-structured interviews explored participants’ experiences | Goals were individualised, and the calendar could be used for personal reminders and appointments |
| [7] Oksnebjerg et al., 2020 | - ReACT App - a calendar   that interacts with the other features, diary notes, contacts, checklists, and memos | Provide support for various aspects of prospective and retrospective memory, and structuring daily activities | - Study team provided participants to access to app when recruited | - Participants accessed a personal user account, and carers could support them via a parallel login - Written materials supported the implementation of the app and detailed the support hotlines | Reminder systems and app preferences could be amended, and carers could view, edit and add information via parallel access |
| [8] Kerssens et al., 2015 | - Companion -Touch screen computer that delivers audio-visual programs constructed of images, music and messages from individuals who are relevant to the user - Provides cues and primes for important tasks, reminders, and reminiscence therapy | Help people with dementia and carers manage common dementia symptoms, promote independence, improve behaviours and overall quality of life, without placing increased burden on carers | - Trained care specialist created a menu of relevant programs for each recipient - 2 researchers provided initial setup and guidance on use | - During a care needs interview, carers picked 1-4 goals - Intervention - carers given diaries to facilitate use of intervention and called weekly for updates - Post-intervention technology adoption questionnaire and semi-structured interview were completed. Carers also rated how the person with dementia was doing regarding the goals | Personalised to individual households and recipients through a life story and care needs interview |
| [9] McGoldrick et al., 2019 | - Free to download dementia app for smartphones and tablets - Includes a ‘’Reminder’’ tool which sends alerts about an event, prompting the user to remember it | Support people to improve their self-management skills and maintain their independence by enabling them to remember important activities, such as taking medication, to decrease reliance on carers | - Primary researcher conducted initial interview, baseline assessments and met with participants during intervention | - Initial interview identified target behaviours and aid use - Intervention - researcher met with participants weekly to decide upon targets, set reminders, and for further app training. Weekly form listing individual memory targets and completion times was provided to partners. When no target could be identified, the researcher set a reminder for participants to text or phone the researcher. Form was used daily to record whether activities were remembered and completed | Personal tablets/smartphone used, and prospective memory targets personalised |
| [10] Kerkhof et al., 2019 | - FindMyApps - User profile, ability to choose main and sub-categories of app interest, pages of recommended apps and specific app information | Help people with mild dementia to select relevant apps that meet their needs, encourage self-management and meaningful activities, and contribute to better quality of life | - Research team and development company | - Prototypes installed on tablets. Users were presented with scenarios which encouraged them to navigate the app. Research team observed use behaviour through videos and interviews | Ability to navigate preferred activity apps |
| [11] Boman et al., 2014 | - Touch screen computer with camera, headset, and contact pictures | Support people with dementia to make calls independently, stay in contact with their social network and prevent isolation | - First and second authors responsible for leading intervention sessions and set-up | - Interviews and observations at participants’ homes - Intervention – sessions conducted in a living laboratory, lasted 2-3hrs (including a break), and were filmed. Participants were shown the features of the videophone and asked to carry out 3 tasks: to make a call to their significant other, to answer a call from their significant other, and make a call to the mock-up emergency services. - After, participants were interviewed about the functions and usability of the videophone | The images of people to call could be personalised to relevant contacts |
