# Supplementary material for: Exploring the Role of Web-Based Interventions in the Self-management of Dementia: Systematic Review and Narrative Synthesis
Source: J Med Internet Res. 2021 Jul 26;23(7):e26551. doi: 10.2196/26551 (PMC8367157; doi:10.2196/26551)
Supplement: Multimedia Appendix 3 [file jmir_v23i7e26551_app3.docx]

Table S3. Outcome measures and key findings from each included study.

| *Study* | *Outcome Measures* | *Key Findings* |
| --- | --- | --- |
|  |  |  |
| **[1]** Perilli et al., 2012 | *Quantitative – Mean number of independent phone calls:* In each session, the RA recorded the total number of phone calls and whether they were made independently.  *Mean number of calls answered by the target partners:* In each session, the number of phone calls which were met with the answer of the target partner were recorded.  *Mean call time:* RA recorded conversation time. | *Independent phone calls:* At baseline, no independent phone calls made. During intervention, 2 participants had a mean of 3 calls per session, and the other 2 had a mean of 4 per session.  *Calls answered by partners:* 2 participants had a mean of 2 calls per session and 2 had a mean of 3 calls.  *Call time:* 2 participants had a mean conversation time of about 5 mins per session, one was about 6 mins, and one was just over 5 mins.  Suggests that the system was effective in helping people with AD make independent phone calls. |
| **[2]** Perilli et al., 2013 | *Quantitative – Mean number of independent phone calls:* In each session, the RA recorded the total number of phone calls and whether they were made independently.  *Mean number of calls answered by the target partners:* In each session, the number of phone calls which were met with the answer of the target partner were recorded.  *Mean call time:* RA recorded conversation time.  *Social validation assessment:* 35 healthcare professionals rated the performance of one of the five participants after watching two 3-min video recordings: one with a standard phone device, and one with the intervention. Rating was carried out through a five-item questionnaire, where 5 was the best rating. | *Independent phone calls:* At baseline, no independent phone calls made. During the intervention, there was an overall mean of nearly four independent phone calls per session.  *Calls answered by partners:* Overall mean was between approximately two and a half and three.  *Call time:* Overall mean conversation time per session was about 7 min.  *Social validation assessment:* Mean scores for the five items of the questionnaire varied between 3.80 and 4.63.  Extends the evidence available on the intervention used to enable people with AD to make phone calls independently and successfully. Social validation data was supportive of the computer-aided system and underlined its value in enhancing the independence, comfortableness, and social image of participants, and its overall practicality and usefulness in daily contexts. |
| **[3]** Lancioni et al., 2017 | *Quantitative – Mean percentage of activities:* The number of activities the participant started as scheduled within first baseline and intervention phase.  *Mean number of steps:* The number of steps participants performed correctly for the activities started in the second baseline and intervention phase. | *Mean percentage of activities:* During first baseline phase, participants’ mean percentages were between 0-14. During the intervention, the participants’ mean percentages of activities started independently were close to 100. The participants responded to al the technology-regulated reminders or missed only very few of them.  *Mean number of steps:* During second baseline phase, participants’ mean percentages were always below 35. In the intervention, the percentages of correct steps were near or above 90 for all participants.  The use of a technology-aided program appears to be an effective resource with multiple practical implications. The program’s components were suitable to positively engage participants. |
| **[4]** Lancioni et al., 2018 | *Study 1*  *Quantitative – Mean percentage of activities started independently:* At baseline, the RA read a list of 5 or 6 activities and the times at which they were due and placed the list on the table. The number of scheduled activities the participant started independently was noted. During intervention session, participants were provided with the technology, which promoted independent start of the activities.  *Mean percentage of activity steps carried out correctly per session:* At baseline, the RA asked the participant to carry out 5 or 6 activities to determine how many steps were carried out correctly. For intervention session, the participant was provided with the technology, which promoted correct performance of the activities.  *Study 2*  *Quantitative – Mean frequencies of step responses:* During baseline, participants were provided with the technology and walker, but received no prompts or stimulation. During intervention, prompts and stimulation were added.  *Mean percentages of positive involvement per session:* Positive involvement was classed as singing, positive verbalisations and smiles etc. and recorded through observations. | *Study 1*  *Mean percentage of activities started independently –* At baseline, the participants’ mean percentage was 0. During intervention this was (close to) 100. Participants responded to all reminders or missed a few.  *Mean percentage of activity steps carried out correctly per session –* At baseline, the mean percentage of correct steps was always below 40. During intervention, the overall mean percentages were near or above 90. The differences between baseline and intervention data were statistically significant for all participants (*P*<.01 – *P*<.05).  *Study 2*  *Mean frequencies of step responses:* During baseline, participants head mean frequencies per session below 50. During intervention, the mean frequencies per session increased to between about 100 and over 150.  *Mean percentages of positive involvement per session:* During baseline, the mean was between 0 and 8. During intervention, the mean percentages ranged from below 10 to near 80. The differences between baseline and intervention were statistically significant *(P* < .01) for all participants on step responses and for 6 participants on signs of positive involvement. |
| **[5]** Lancioni et al., 2019 | *Quantitative – Mean frequencies of correct target responses:* 3 was the maximum frequency possible as each session provided the participant with the opportunity to bring 3 objects to 3 destinations.  *Mean frequencies of intervals with indices of enjoyment/appreciation:* 4 was the maximum frequency possible as each session contained 4 observation intervals in which the presence or absence of the measure was recorded. | *Target responses:* At baseline, the mean frequencies of correct responses were between 0-1, over 4 sessions. During intervention, it was between 2.8-3 per session, and an overall mean across participants of 2.9 (3 was the maximum).  *Intervals of enjoyment/appreciation:* Baseline mean frequencies of intervals with indices of enjoyment/appreciation ranged from 0-1. During the intervention, the mean ranged from 2.5-3.5 per session, and an overall mean across participants of 3 (4 was the maximum).  The intervention was highly effective in improving overall performance in goal-directed walking in AD. |
| **[6]** Thorpe et al., 2019 | *Quantitative – Activity levels:* Data from devices that calculated activity time  *Self-reported activity, mobility and goal attainment:* Questionnaires completed pre-and post-study regarding mobility, activity, caregiver burden, functional performance and quality of life. Mobile self-reports were issued daily on a 5-point scale (much less than normal – much more than normal)  *Qualitative – Interviews:* Semi-structured, at the end of the study, to explore experiences and outcomes. | *Activity levels:* Use of a smartphone and smartwatch were adequate in helping monitor activity levels.  *Self-reported activity, mobility and goal attainment:* 2 participants who were satisfied with their current lifestyle followed goals to maintain their schedule. Participants also found it difficult to recall their goal over the duration of the study.  *Interviews:* the support offered by smart technology addressed functional, memory, safety, leisure and psychosocial needs. 4 participants perceived this support to positively impact their health, mostly regarding motivation to be active, with one participant further describing considerable impact on anxiety, independence, activity and caregiver burden.  The findings suggest the potential impact of smartphones and wearable devices to offer support for people with dementia in their everyday life. |
| **[7]** Øksnebjerg et al., 2020 | *Quantitative – Log data:* App usage for all participants and caregivers for a maximum of 90 days.  *Survey:* A web-based survey to collect additional background information and feedback on the app. It was distributed via email 3 to 4 months after inclusion in the study. In cases where email correspondence was unsuccessful, a printed version of the survey was sent out by mail. Two versions of the survey were distributed: one for participants and another by-proxy version for carers.  *USEdem:* For participants who had activated the app, the questionnaire was included in the survey, and a by-proxy version was delivered to carers. This modified version contains 12 items and was adapted to be applied to people with dementia. Scores on each item range from 1 to 5 on a Likert scale, with a total score between 12 and 60, higher scores indicating higher ratings.  *Qualitative - Survey:* Data from the survey feedback on reasons for not using the app, were processed and summarized in themes, as outlined in constant comparison analysis. | *Adoption:* Adoption of the app was defined as a minimum period of 90 days between the first and last use of the app. 18 participants and 7 of the carers became adopters. Overall, 47 participants and 78 carers never activated the app. Reasons for not using included needing to learn to use it and forgetting to use it.  *USEdem:* An overall average score of 40 (range 21-55) for participants and 34 (range 18-51) for carers, which indicated a generally positive rating of the app with regard to usefulness, satisfaction, and ease of use, but with large variation.  *Surveys:* Data revealed that there were no significant differences between adopters and nonadopters when it came to how much experience they had using a tablet, their skills when using it, and how much help they needed to use it.  For participants who became adopters, the ReACT app and the methods for self-applied implementation were applicable. However, the results were also in accordance with the well-known challenges of non-adoption and nonadherence to digital health interventions. It underlined the personal and contextual factors that influence adoption. These factors need to be considered when designing and implementing digital interventions for people with dementia. |
| **[8]** Kerssens et al., 2015 | *Qualitative – Goal and subjective attainment*  *Quantitative – Technology implementation and feasibility, Goals and subjective attainment:* Carers asked to rate how the person with dementia was doing with goals with Better, Stable, Worse, N/A, and the level of functioning post-intervention with Much less than expected, Somewhat less than expected, As expected, Somewhat more than expected.  **Technology adoption:** People with dementia answered Yes/No, carer answer options ranged from Very True to Very Untrue**.** | *Goals and subjective attainment:* 11 out of 25 goals were as expected or better than expected, whereas 8 goals were less than expected. Barriers to use included people with dementias’ inability to use the intervention and ignoring the intervention even when they perceived them as positive.  *Technology implementation and feasibility:* All participants accepted the intervention. 5 out of 7 couples kept the *Companion* post-intervention.  *Technology Adoption:* People with dementia perceived many aspects of the intervention positively and helped them relax and enjoy life. Carers indicated that they valued the intervention.  The intervention facilitated meaningful and positive engagement in the home and helped people with dementia and carers cope with symptoms and needs in daily life. |
| **[9]** McGoldrick et al., 2019 | *Qualitative – Usefulness, strengths, limitations, and future use*    *Quantitative – Unified Theory of Acceptance and Use of Technology Questionnaire:* Completed pre-and post-study by 2 participants in the interviews. Questionnaire concerned eight domains, such as attitudes towards the technology, and behavioural intentions, and each item was scored on a scale of 1-5. Items pooled to give overall domain score.  *Target events remembered:* Frequencies calculated for the percentage of target events remembered out of all events for each week. | *Qualitative:* Positive overall impression of the app. Gave participants a sense of independence. Difficulties with the app and lack of insight contributed to the one participant withdrawing from intervention phase.  *UTAUT:* Participant FD had an overall decrease in pre/post scores(positive), but the mean score for anxiety domain increased. Participant SI mean scores increased in 4 domains(negative). SI wanted to continue to use the app, but unsure about helpfulness of app, as they were learning to use it independently.  *Target events remembered:* Participant FD completed 49% of tasks during baseline and 93% during intervention. Participant SI completed 69% during baseline and 95% during intervention. Participant CE completed 51% across 11-weeks of baseline.  The evidence supports the effectiveness of MindMate in reducing prospective memory problems. Some concerns were raised about technical difficulties and frustration with use. 2 participants expressed intention for continued use. |
| **[10]** Kerkhof et al., 2019 | *Qualitative – Behavioural observation in interviews and scenario testing of intervention* | User insight provided valuable knowledge to develop a workable app. Major insight was that the user-interface elements, such as pages and button, had to be simple and logically integrated to support users in operating and understanding the tool.  FindMyApps selection tool makes a unique contribution to the field of dementia. It can support people with mild dementia in using the relevant apps that will contribute to a better quality of life. |
| **[11]** Boman et al., 2014 | *Qualitative – Interviews and observations:* Conducted with participants and their significant others at their homes. Interview focused on participants’ experiences of using phones, computer, and Skype, and how they responded to any difficulties. Participants then observed using their own technology, scored on a 3-point scale (3=no difficulty, 2=minor difficulty, 1=major difficulty).  *Intervention sessions:* Observations and interviews were used to examine the usability of the mock-up. Questions were asked on satisfaction of design and how easy the features were to understand and use when carrying out 3 set tasks**.** Participants were observed when using the mock-up to carry out tasks. | *Interviews and observations:* Some participants were quite active with using a mobile or telephone, but some reported avoiding using the phone unless necessary and problems with remembering numbers. All participants could identify their phones and/or computers with no difficulty (one was minor when identifying computer). However, they struggled when performing the correct actions and choosing the correct buttons when calling or receiving a call.  *Intervention sessions:* Observations showed that initially most participants did not know how to make, or answer calls on the mock-up. However, after some feedback all could carry out the tasks independently. Participants perceived the mock-up as enjoyable to use and would have like to have it in their homes. However, they would have like to adjust the features of the video phone to their individual needs and wishes.  The findings suggest that the videophone mock-up was enjoyable to use and effective in enabling people with dementia to make calls independently. The difficulties with phones and computers observed in participants’ homes were not present when using the mock-up. |
